# Effects of monocyte-endothelium interactions on the expression of type IV collagenases in monocytes

**DOI:** 10.3892/etm.2014.2109

**Published:** 2014-12-05

**Authors:** YONG-QIN LI, RUI LIU, JIA-HONG XUE, YAN ZHANG, DENG-FENG GAO, XIAO-SAN WU, CONG-XIA WANG, YU-BAI YANG

**Affiliations:** 1 Department of Cardiology, The Second Hospital of Xi’an Jiaotong University, Xi’an, Shaanxi 710004, P.R. China; 2 Department of Physiology and Pathophysiology, Medical School of Xi’an Jiaotong University, Xi’an, Shaanxi 710061, P.R. China

**Keywords:** atherosclerosis, collagenase IV, monocyte, endothelium, tumor necrosis factor-α, interleukin-1β

## Abstract

The adhesion of monocytes to endothelial cells is one of the early stages in the development of atherosclerosis. The expression of type IV collagenases, which include matrix metalloproteinase (MMP)-2 and MMP-9, in monocytes is hypothesized to play an important role in monocyte infiltration and transformation into foam cells. The aim of the present study was to examine the effects of monocyte-endothelium interactions on the expression levels of type IV collagenases and their specific inhibitors in monocytes, and to investigate the roles of tumor necrosis factor (TNF)-α and interleukin (IL)-1β in this process. Monocytes were single-cultured or co-cultured with endothelial cells. The expression of the type IV collagenases, MMP-2 and MMP-9, and their specific inhibitors, tissue inhibitor of metalloproteinase (TIMP)-1 and TIMP-2, in monocytes was determined by immunohistochemistry followed by image analysis. The expression levels of MMP-2 and MMP-9 were found to be low in the single-culture monocytes, but increased significantly when the monocytes and endothelial cells were co-cultured. However, treatment with monoclonal TNF-α or IL-1β antibodies partially inhibited the upregulated expression of MMP-2 and MMP-9 in the co-cultured monocytes. Expression of TIMP-1 and TIMP-2 was observed in the single monocyte culture, and a small increase in the expression levels was observed when the monocytes were co-cultured with endothelial cells. Therefore, monocyte-endothlium interactions were shown to increase the expression of type IV collagenases in monocytes, resulting in the loss of balance between MMP-2 and -9 with TIMP-1 and -2. In addition, TNF-α and IL-1β were demonstrated to play important roles in this process.

## Introduction

Matrix metalloproteinase (MMP) is a general term for the group of proteinases that degrade the extracellular matrix (ECM). Type IV collagenases consists of two types of MMP, 72-kDa MMP-2 and 92-kDa MMP-9, which are both synthesized and secreted by neutrophils and macrophages. The two MMPs are able to degrade the ECM in the basement membrane. Among multiple types of MMPs, type IV collagenase is closely associated with diabetes and cardiovascular disease (1–3). The substrates of type IV collagenases include type IV and V collagens, fibronectin, laminin, elastin and denatured collagen matrix (4,5). The activity of MMPs can be inhibited by the corresponding tissue inhibitors of metalloproteinases (TIMPs). TIMPs generate MMP-TIMP complexes through the combination of cysteine residues in the N-terminus functional region with the active center of the MMP, thereby blocking the ability of the MMP to bind to substrates. Four types of TIMP have been identified and are referred to as TIMP-1, -2, -3 and -4. The specific inhibitors of the type IV collagenases, MMP-2 and MMP-9, are TIMP-2 and TIMP-1, respectively. By adjusting the relative concentrations of MMPs and their inhibitors, the body is able to control the composition of the ECM (6).

Increasing experimental evidence has revealed that changes in the expression of type IV collagenases and the corresponding inhibitors, and subsequently the disproportion of the two, play an important role in the occurrence and development of atherosclerosis and vascular restenosis (7,8). The detection of MMP expression in normal human arterial tissue and atherosclerotic plaque tissue demonstrates that type IV collagenases, TIMP-1 and TIMP-2 are expressed in normal smooth muscle cells, but are inactive. However, the increase in type IV collagenases in the smooth muscle cells and macrophages present in atherosclerotic plaques indicates their matrix-degrading activity (3). In addition, endothelial cells covering the plaque surface, which differ from endothelial cells in other areas of the body, are able to express active type IV collagenases (9). In newly isolated monocytes, the expression of MMP-9 is low; however, a series of cytokines found in atherosclerotic plaques, including interleukin (IL)-1β and tumor necrosis factor (TNF)-α, are able to increase the expression and secretion of MMP-9 (10–12). MMP-2 and MMP-9 are considered to be the principal MMPs secreted by monocytes that are capable of degrading type IV collagen, the main constituent of the basement membrane (13). Furthermore, the expression of MMP-2 and MMP-9 in monocytes is particularly important in penetrating the first basement membrane barrier of the ECM in the development of atherosclerosis. Thus, factors that regulate the expression of MMP-2 and MMP-9 in monocytes may affect the process of atherosclerosis development.

Monocyte adhesion to endothelial cells and their subsequent infiltration play an important role in the early onset of atherosclerosis (14). The contact adhesion of the two cells is not solely physical and there is often an interactive dialogue between the cells. In the contact process, a number of corresponding signaling transduction pathways are triggered that significantly affect the cell phenotype and function, including the expression of MMPs (15). For instance, the interaction of T lymphocytes with endothelial cells may increase the expression of MMP-2, which is dependent on the mediation of vascular cell adhesion molecules expressed in the endothelial cells (16). Lee *et al* demonstrated that the interaction between monocytes and smooth muscle cells may induce the expression of MMP-1 and MMP-3 (17). Furthermore, Amorino and Hoover observed that the direct contact of monocytes with formalin-fixed human monolayer endothelial cells resulted in an increased expression of MMP-9 (18). However, in these studies, the precise mechanism of interaction between the monocytes and endothelial cells that caused the increase in MMP expression was not studied in depth. In addition, the effect of the interaction between monocytes and endothelial cells on the expression levels of type IV collagenases and their specific inhibitors in monocytes remains unknown.

In the present study, single and mixed cultures of monocytes and endothelial cells were established, and changes in the expression levels of the type IV collagenases, MMP-2 and MMP-9, as well as their specific inhibitors, TIMP-1 and TIMP-2, were investigated in the monocytes.

## Materials and methods

### Cell culture

A monocyte cell line (U937) and human umbilical vein endothelial cells (HUVECs) were obtained from the National Infrastructure of Cell Line Resources (Beijing Union Medical College, Beijing, China). The cells were maintained in RPMI 1640 medium (Gibco Life Technologies, Carlsbad, CA, USA) supplemented with 10% calf serum (Huamei Bioengineering Co. Ltd., Shanghai, China), 20 mM sodium bicarbonate (Sigma-Aldrich, St. Louis, MO, USA) and 1% penicillin/streptomycin mix (Invitrogen Life Technologies, Carlsbad, CA, USA). The cells were incubated at 37°C in a 5% CO_2_ incubator.

### Grouping

Six experimental groups were established as follows: Endothelial cell and monocyte co-culture group; co-culture group supplemented with TNF-α monoclonal antibodies (2 μg/ml); co-culture group supplemented with IL-1β monoclonal antibodies (2 μg/ml); co-culture group supplemented with TNF-α (2 μg/ml) and IL-1β (2 μg/ml) monoclonal antibodies; single-culture monocyte group; and cultured monocyte group supplemented with conditioned medium from the 12 h co-culture of monocytes and endothelial cells. Each group was cultured serum-free for 24 h post-treatment and subsequently centrifuged at 800 × g for 3 min at room temperature (20–22°C). Immunocytochemical staining was performed on the monocytes. In the five wells of each group, four smears were placed in each well, which were immunocytochemically stained with monoclonal antibodies against MMP-2, MMP-9, TIMP-1 and TIMP-2. The MMP-2, MMP-9, TIMP-1, TIMP-2, TNF-α and IL-1β monoclonal antibodies were purchased from Shanghai SenXiong Biotech Industry Co., Ltd (Shanghai, China).

### Immunocytochemistry and image analysis

Monocytes were centrifuged at 500 × g to remove the medium, washed and centrifuged twice at 500 × g at room temperature, with phosphate-buffered saline (PBS). A monocyte smear was made on the carrier plate, which was subsequently dried in shade for 15 min and slowly placed in 4% paraformaldehyde solution for fixation. Staining was performed using a streptavidin-biotin complex enzyme immunoassay kit (Wuhan Boster Biological Technology, Ltd, Wuhan, China), according to the manufacturer’s instructions. Cells with yellow, brownish-yellow or chocolate-brown colored particles were considered to be positive cells. QWin image processing software (Leica Camera AG, Solms, Germany) was used for image analysis. A view field was randomly selected from each plate. Based on the number of cells, a total of 30–50 cells were selected and their average optical density was measured. The average optical density of the five wells was subsequently calculated. The differences in the optical density values reflected the differences in the color shades, and also the different concentrations of the tested substances.

### Statistical analysis

Experimental data are presented as the mean ± standard deviation. Intergroup data processing was based on the results of the homogeneity test of variance, and the Student-Newman-Keuls test was used to analyze the differences between the groups. P<0.05 was considered to indicate a statistically significant difference. All statistical analyses were performed using SPSS 17.0 statistical software (SPSS, Inc., Chicago, IL, USA).

## Results

### Effect of the different culture conditions on MMP-2 expression

**Figure 1:**
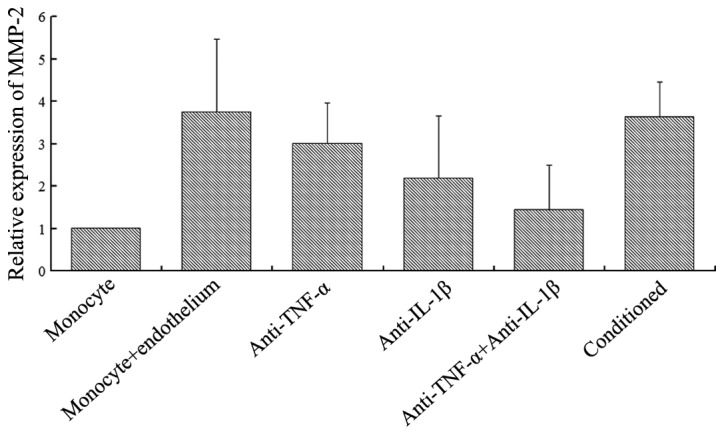
Effect of different culture conditions on MMP-2 expression in monocytes. No statistically significant difference was observed between the conditioned cultured group and the monocyte + endothelium co-cultured group (P>0.05). Statistically significant differences were observed when comparing the conditioned group with the cytokine antibody groups (P<0.05). TNF, tumor necrosis factor; IL, interleukin; MMP, matrix metalloproteinase.

As shown in Fig. 1, a degree of MMP-2 expression was observed when the monocytes were single-cultured; however, MMP-2 expression significantly increased when the monocytes were co-cultured with endothelial cells. In the co-cultured group, the addition of TNF-α (2 μg/ml) or IL-1β (2 μg/ml) monoclonal antibodies reduced MMP-2 expression, while the addition of the two cytokine antibodies significantly reduced the expression of MMP-2. When monocytes were added to the conditioned medium from the co-culture of monocytes and endothelial cells, MMP-2 expression increased. A statistically significant difference was not observed when compared with the two-cell co-cultured group (P>0.05). However, statistically significant differences were observed when comparing the conditioned group with the cytokine antibody groups (P<0.05).

### Effect of the different culture conditions on MMP-9 expression

**Figure 2:**
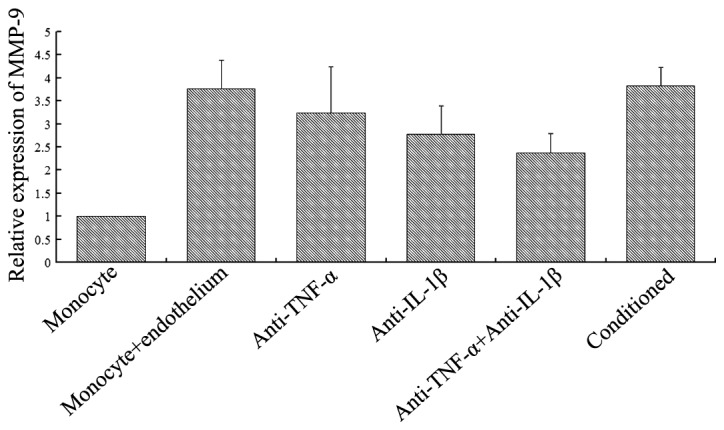
Effect of different culture conditions on MMP-9 expression in monocytes. No statistically significant difference was observed between the conditioned cultured group and the monocyte + endothelium co-cultured group (P>0.05). Statistically significant differences were observed when comparing the conditioned group with the cytokine antibody groups (P<0.05). TNF, tumor necrosis factor; IL, interleukin; MMP, matrix metalloproteinase.

As shown in Fig. 2, the single culture of monocytes exhibited a certain degree of MMP-9 expression. Following the co-culture of monocytes with endothelial cells, the expression level of MMP-9 was significantly higher compared with the single culture. TNF-α (2 μg/ml) or IL-1β (2 μg/ml) monoclonal antibodies were able to inhibit the upregulation of MMP-9 expression, to a certain extent, observed in the two-cell co-culture group. However, the addition of the two cytokine antibodies did not fully inhibit the upregulation of MMP-9 expression, although the level was significantly reduced compared with the two-cell co-culture group. When monocytes were added to the conditioned medium from the co-culture of monocytes and endothelial cells, the MMP-9 expression increased, and the difference with the two-cell co-cultured group was not significantly different (P>0.05). However, statistically significant differences were observed when comparing the conditioned group with the cytokine antibody groups (P<0.05).

### Effect of the different culture conditions on TIMP-1 expression

**Figure 3:**
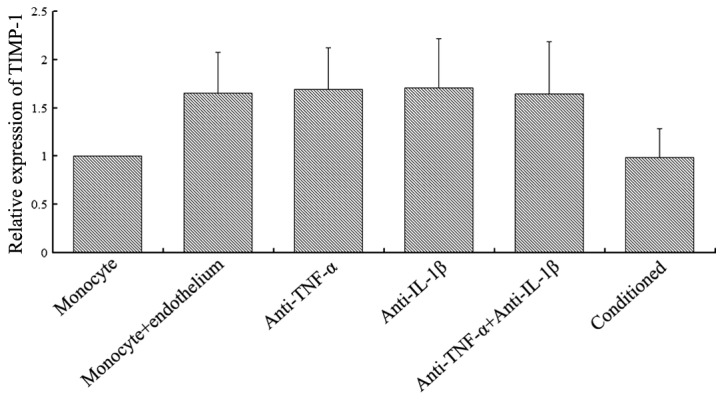
Effect of different culture conditions on TIMP-1 expression in monocytes. No statistically significant difference was observed between the monocyte single-cultured group and the conditioned cultured group (P>0.05). Statistically significant differences were observed when comparing the monocyte single-cultured group with the monocyte + endothelium co-cultured groups (P<0.05). No statistically significant differences were observed when comparing the monocyte + endothelium co-cultured group with the cytokine antibody groups (P>0.05). TNF, tumor necrosis factor; IL, interleukin; TIMP, tissue inhibitor of metalloproteinase.

As shown in Fig. 3, TIMP-1 expression was observed in the monocyte single-culture. When the monocytes were co-cultured with endothelial cells, the expression levels of TIMP-1 increased significantly (P<0.05), but the enhanced amplitude was less than two times the monocyte single-culture. The addition of TNF-α (2 μg/ml) and/or IL-1β (2 μg/ml) monoclonal antibodies to the co-cultured group revealed no effect on TIMP-1 expression (P>0.05). Furthermore, the addition of conditioned medium from the co-culture of endothelial cells and monocytes to the cultured monocytes demonstrated no effect on TIMP-1 expression when compared with the single-culture monocytes (P>0.05).

### Effect of the different culture conditions on TIMP-2 expression

**Figure 4:**
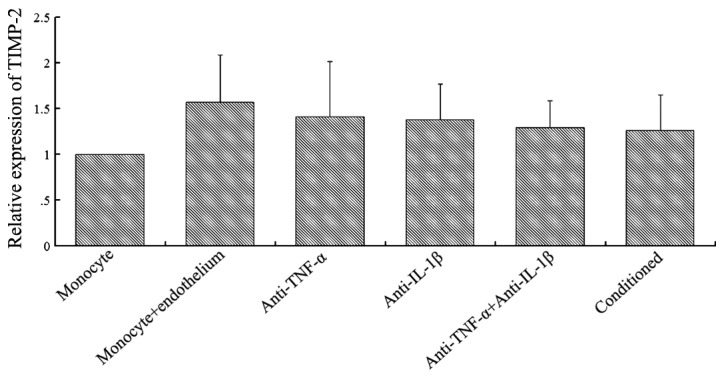
Effect of different culture conditions on TIMP-2 expression in monocytes. Statistically significant differences were observed when comparing the monocyte single-cultured group with the conditioned cultured and monocyte + endothelium co-cultured groups (P<0.05), and when comparing the monocyte + endothelium co-cultured group with the cytokine antibody groups (P<0.05). A significant difference was also observed between the conditioned cultured group and the monocyte + endothelium co-cultured group (P<0.05). TNF, tumor necrosis factor; IL, interleukin; TIMP, tissue inhibitor of metalloproteinase.

As shown in Fig. 4, TIMP-2 was expressed when the monocytes were single-cultured, but a significantly increased expression was observed when the monocytes were co-cultured with endothelial cells (P<0.05). The addition of TNF-α (2 μg/ml) or IL-1β (2 μg/ml) monoclonal antibodies to the co-culture group partially inhibited the upregulation of TIMP-2 expression observed in the two-cell co-culture group. However, simultaneous addition of the two cytokine antibodies did not completely inhibit the upregulation of TIMP-2 expression. The addition of conditioned medium from the co-culture of endothelial cells and monocytes to the cultured monocytes caused an increase in TIMP-2 expression when compared with the single-cultured monocytes (P<0.05). A significant difference was also observed between the conditioned cultured group and the monocyte-endothelium co-cultured group (P<0.05; n=5).

## Discussion

The adhesion of monocytes to endothelial cells and secondary subendothelial migration play important roles in the development of atherosclerosis. Furthermore, monocyte regulation of type IV collagenase expression is particularly important for the decomposition of the ECM during infiltration (19). However, there have been no definite results on whether the adhesion of endothelial cells and monocytes has an effect on the expression levels of type IV collagenases and their specific inhibitors. The present study observed that when monocytes were cultured alone, the type IV collagenases MMP-2 and MMP-9, and their specific inhibitors, TIMP-1 and TIMP-2, were expressed to a certain extent. However, following co-culture with endothelial cells, the expression levels of type IV collagenases were significantly increased, while the expression levels of their specific inhibitors increased to a lesser degree. Following the adhesion of monocytes to endothelial cells, a functional change occurs where the ability to decompose the matrix becomes stronger. This change is beneficial to the migration of the monocytes and their ingestion of the lipids in the arterial wall, which causes the transformation into foam cells. The current experiments also demonstrated that TNF-α and IL-1β partially mediated the expression levels of the monocyte type IV collagenases, MMP-2 and MMP-9. These observations indicate that the functional change of type IV collagenases in monocytes during the adhesion of monocytes to endothelial cells plays an important role in the occurrence of atherosclerosis. Thus, the regulation of MMP expression may become an intervention target in the treatment of atherosclerotic vascular disease (19,20).

In atherosclerotic vascular disease caused by diabetes and hypertension, an important part of the pathogenesis process is the aggregation of leukocytes in the circulatory system and their adherence locally in endothelial cells through adhesion molecules (21). Following adhesion, the leukocytes penetrate the endothelial cells and the corresponding basement membrane; however, the exact mechanism remains unclear. A previous study indicated that MMPs facilitate this process (1). The direct contact of cytokine-stimulated monocytes with the human endothelial cell monolayer has been shown to result in increased MMP-9 expression in monocytes (22); however, the precise mechanism is yet to be elucidated. During the infiltration of the artery wall by inflammatory cells, the degradation of the basement membrane of the endothelial cells by MMPs damages the endothelial barrier function, resulting in an increase in the inflow of plasma proteins. Once the inflammatory cells in the vessel wall are infiltrated, they are able to interact with the ECM and oxidized low-density lipoproteins, further promoting the expression of MMPs in macrophages (23). Macrophages are able to secrete stimulatory factors that induce the secretion of MMPs and promote their activation. In the development of atherosclerosis, the increased activity of MMPs leads to changes in the structure of the vessel wall, resulting in the remodeling of the blood vessel wall (23,24). Thus, the effective mechanism of the monocyte-endothelium interaction on MMP expression in monocytes is of great significance for the study of atherosclerosis incidence.

The present study observed that when monocytes were cultured alone, the expression levels of MMP-2 and MMP-9 were low, while the expression levels of TIMP-1 and TIMP-2 were relatively high. This observation indicates that for type IV collagenases, under normal conditions, the potentially stronger control mechanism of TIMPs is able to regulate the activity of the enzymes. Since MMPs have numerous degradable substrates and are able to degrade a wide range of ECM proteins, such as collagen, elastin and glycoproteins, controlling the activity of MMPs under physiological conditions is important. In monocytes, the basal expression levels of TIMP-1 and TIMP-2 are high and the potential secretion is large, which is beneficial to achieving the timely control of MMPs and maintaining a stable organizational structure under physiological conditions. Under certain pathological conditions, the expression levels of MMPs and TIMPs are often in disorder. For example, during the occurrence and development of atherosclerosis, smooth muscle cells proliferate and migrate to the tunica intima, forcing the cells to pass through the ECM of the vessel wall and the basement membrane. Previous experiments have demonstrated that the upregulation of MMP expression is closely associated with the migration of smooth muscle cells (3). Thus, the use of MMP inhibitors may significantly reduce the migration and proliferation of smooth muscle cells (25).

In the early stage of atherosclerosis development, a number of monocytes penetrate the vascular endothelium to infiltrate the vessel wall. During the process, the basement membrane and the blood vessel walls must be penetrated. Therefore, the expression of MMPs in monocytes, particularly the expression of the type IV collagenases, which are able to degrade type IV collagen in the basement membrane, plays an important role in the onset of atherosclerosis. Hojo *et al* (26), Matías-Román *et al* (27) and Mostafa Mtairag *et al* (28) studied the effects of the monocyte-endothelium interaction on MMP secretion and confirmed that co-culture of the two cells promoted the expression of MMPs in leukocytes; however, the studies did not conduct a thorough investigation of the specific mechanisms underlying this process. The experimental results of the current study are consistent with the conclusions of the aforementioned studies. Furthermore, the present study conducted an in-depth investigation on the underlying mechanism, and revealed that TNF-α and IL-1β play important roles. However, these cytokines are not the only stimulatory factors, as the addition of TNF-α and IL-1β antibodies did not completely inhibit the increase in the expression levels of MMP-2 and MMP-9, indicating that a number of other cytokines are involved in the endothelial cell-stimulated upregulation of MMP-2 and MMP-9 expression in monocytes.

With regard to whether endothelial cell and monocyte adhesion may directly activate the gene signaling transduction pathways of type IV collagenases and their inhibitors by adhesion molecules, the present study revealed that in the regulation of MMP-2 and MMP-9, this direct activation pathway may not exist or that such a direct regulatory role is very small. However, for TIMP-1 and TIMP-2, this direct activation process may exist. When compared with the conditioned culture group, in the monocytes of the two-cell co-culture group, relatively high expression levels of TIMP-1 and TIMP-2 were observed. This increase in the expression levels of the two types of inhibitor may be due to the contact of the two cells directly inducing the expression of TIMP-1 and TIMP-2. Through the corresponding combination of adhesion molecules with their ligands, the signaling transduction pathways of TIMP-1 and TIMP-2 genes may be activated, causing an increase in expression levels. The current study revealed that when compared with the single-cultured monocyte group, the expression levels of MMP-2 and MMP-9 in the conditioned group were significantly higher. However, when compared with simple co-cultured group, no statistically significant difference was observed, indicating that the increased secretion of type IV collagenases caused by the contact adhesion of the endothelial cells and monocytes plays a major role in affecting the expression levels of MMP-2 and MMP-9 in monocytes. The expression levels of MMP-2 and MMP-9 significantly increased following exposure of the monocytes to endothelial cells. However, the expression level increases in the corresponding inhibitors, TIMP-1 and TIMP-2, were lower. This observations indicates that under physiological conditions, TIMP-1 and TIMP-2 are able to regulate MMP activity; however, during the contact adhesion of endothelial cells and monocytes, the upregulation of MMPs and their inhibitors is not synchronized. The original dominant position of the TIMPs has changed, and the balance between the two has been lost, which may be deduced from the reduced expression levels of the inhibitors. Subsequently, the expression and activity of type IV collagenases increase and the ability to decompose the basement membrane collagen and vascular wall matrix is enhanced.

In conclusion, the present study revealed that the interaction of monocytes and endothelial cells may stimulate the functional transformation of monocytes, resulting in an increase in the expression levels of the type IV collagenases, MMP-2 and MMP-9, and the destruction of the balance between them and their specific TIMPs. The interaction of monocytes and endothelial cells induces functional changes in the monocytes, which contribute to the occurrence and development of atherosclerosis.
